# O_2_ dynamics in the rhizosphere of young rice plants (*Oryza sativa* L.) as studied by planar optodes

**DOI:** 10.1007/s11104-015-2382-z

**Published:** 2015-02-06

**Authors:** Morten Larsen, Jakob Santner, Eva Oburger, Walter W. Wenzel, Ronnie N. Glud

**Affiliations:** 1 Institute of Biology and Nordic Center for Earth Evolution (NordCEE), University of Southern Denmark, 5320 Odense M, Denmark; 2 Scottish Marine Institute, Scottish Association for Marine Science, Oban, Scotland PA37 1QA UK; 3 Greenland Climate Research Centre (CO Greenland Institute of National resources), Kivioq 2, Box 570, 3900 Nuuk, Greenland; 4 Arctic Research Centre, Aarhus University, 8000 Aarhus, Denmark; 5 Rhizosphere Ecology and Biogeochemistry Group, Institute of Soil Science, Department of Forest and Soil Sciences, University of Natural Resources and Life Sciences Vienna, 3430 Tulln, Austria

**Keywords:** Planar optode, Rice (*Oryza sativa* L.), Radial oxygen loss, Rhizosphere, Plaques imaging, Oxygen

## Abstract

**Background and aims:**

Radial O_2_ loss (ROL) strongly affect the O_2_ availability in the rhizosphere of rice. The ROL create an oxic zone around the roots, protecting the plant from toxic reduced chemical species and regulates the redox chemistry in the soil. This study investigates the spatio-temporal variability in O_2_ dynamics in the rice rhizosphere.

**Method:**

Applying high-resolution planar optode imaging, we investigated the O_2_ dynamics of plants grown in water saturated soil, as a function of ambient O_2_ level, irradiance and plant development, for submerged and emerged plants.

**Results:**

O_2_ leakage was heterogeneously distributed with zones of intense leakage around roots tips and young developing roots. While the majority of roots exhibited high ROL others remained surrounded by anoxic soil. ROL was affected by ambient O_2_ levels around the plant, as well as irradiance, indicating a direct influence of photosynthetic activity on ROL. At onset of darkness, oxia in the rhizosphere was drastically reduced, but subsequently oxia gradually increased, presumably as root and/or soil respiration declined.

**Conclusion:**

The study demonstrates a high spatio-temporal heterogeneity in rhizosphere O_2_ dynamics and difference in ROL between different parts of the rhizosphere. The work documents that spatio-temporal measurements are important to fully understand and account for the highly variable O_2_ dynamics and associated biogeochemical processes and pathways in the rice rhizosphere.

## Introduction

Due to the importance of rice (*Oryza sativa*) as a staple food source in global human nutrition, it is likely the most studied plant growing in flooded and waterlogged soils. However, studies on O_2_ dynamics in the rice rhizosphere remain a challenge and the biogeochemical functioning of the rice rhizosphere is still rudimentary. Flooded conditions are a widespread phenomenon among rice and it is estimated that about 75 % of all rice are grown under O_2_ depleted conditions in flooded and waterlogged soils, during part or all of the cropping period (Roger et al. 1993). Rice plants growing in O_2_ depleted soils are potentially exposed to high levels of harmful reduced/toxic chemical species, like H_2_S and Fe^2+^ (Armstrong and Armstrong 2005; Mongon et al. 2014). To overcome these challenges a range of wetland plants, including some rice genotypes, have evolved two adaptations (I) development of a barrier against solutes and gasses e.g., by increased suberization in exodermis and/or ligninfication in sclerenchyma cells (Greenway et al. 2006; Kotula et al. 2009) and/or (II) leakage of O_2_ to establish a protective oxygenated zone around the roots, a strategy facilitated by well-developed gas conducting aerenchyma inside the roots (Armstrong 1980; Kirk 1993). The two strategies vary extensively between different species of wetland plants and even among genotypes of the same species of wetland land plants and even among genotypes of the same plant.

The widespread occurrence of ROL in rice plants and the ability to form a barrier to ROL when grown in stagnant solutions is extensively reported e.g., (Colmer et al. 1998; Insalud et al. 2006; Kotula et al. 2009). However, the ability to form a barrier to ROL varies among different genotypes as demonstrated by Colmer et al. (1998) and Colmer (2003), who investigated different rice genotypes of lowland rice from drained and waterlogged soils. The studies demonstrated that genotypes to various extend showed an increase in the root porosity and a development of a tight barrier to ROL, when grown in stagnant O_2_ depleted conditions, as compared to oxygenated conditions (Insalud et al. 2006; Kotula et al. 2009; Shiono et al. 2011). The plasticity in root physiology presumably contributes to the ability of rice to grow in diverse environments at different levels of waterlogging (Colmer 2003).

The O_2_ dynamic associated to roots without a tight barrier to ROL can have important implications for the redox conditions and the biogeochemical processes and pathways that take place in the rhizosphere, such as trace metal availability (Williams et al. 2014) and plaque formations (Mendelssohn et al. 1995). The formation of metal plaque in the rhizosphere of wetland plants is often associated to ROL as plaque mainly reflects the distribution of Fe-oxides (Wu et al. 2012). Plaque formation on roots have been demonstrated to be important for root nutrient and trace metal dynamics, generally limiting phosphorus uptake and acting as sorption sites for trace metal such Arsenic (Jiang et al. 2009; Deng et al. 2010; Seyfferth et al. 2010). Plaque formation on rice roots have generally been studies using extraction based methods (Deng et al. 2010; Cheng et al. 2014) and only few studies have investigated the spatial variation of plaque distributions (Seyfferth et al. 2010), however, the link between spatial variations in ROL and plaque is not well documented.

Traditionally ROL from rice roots have been studied using microelectrodes (Frenzel et al. 1992; Revsbech et al. 1999; Colmer and Pedersen 2008), bulk measurements (Kludze et al. 1993; Kirk and Du 1997) and cylindrical O_2_ cathodes (Armstrong 1994; Insalud et al. 2006). The studies demonstrate that ROL from rice roots can be affected by e.g., irradiance (Waters et al. 1989; Revsbech et al. 1999; Colmer and Pedersen 2008) and flooding (Waters et al. 1989; Pedersen et al. 2009). However, these studies often rely on single point measurements of individual roots or plants grown in artificial medium such as hydroponics or agar medium. Single point measurements have provided valuable insight in root O_2_ dynamics of rice plants, especially in cases where they have been performed on soil grown plants (Frenzel et al. 1992; Revsbech et al. 1999). However they only provide a limited understanding on the overall O_2_ dynamic in the rhizosphere and still little is known about the spatio-temporal variations in ROL of intact rhizosphere grown under natural condition.

Recently, two-dimensional luminescent imaging (planar optodes) have been applied for rhizosphere and soil studies investigating 2D O_2_, pH and pCO_2_ dynamics (Frederiksen and Glud 2006; Blossfeld et al. 2013; Rudolph et al. 2013; Williams et al. 2014). Planar optodes are optical semitransparent sensors that allow real-time and two-dimensional imaging of e.g., O_2_ by means of a luminescent and analyte specific indicator. By imaging the light emitted from the indicator, the analyte concentration can be quantified and related to physical structures in the soil. Planar optodes offer high spatial (>20 μm) and temporal (1–30 s) resolution comparable to those of microelectrodes, but can resolve dynamics of larger areas of the soil rhizosphere (>20 cm^2^) as compared to a single-point measurement (Frederiksen and Glud 2006; Rudolph et al. 2013; Jovanovic et al. 2015). The use of planar optodes has provided new conceptual and quantitative understanding of solute dynamics in such environments and on the importance of microniche structure for the biogeochemical functioning in such environments (Jensen et al. 2005; Schreiber et al. 2012; Faget et al. 2013).

Here we apply two-dimensional, high-resolution planar optode imaging to investigate the spatio-temporal variability in O_2_ dynamics between individual roots and different parts of the root system of rice exposed to different environmental conditions. We furthermore demonstrate a simple system for plaque imaging. Plants were grown in flooded soil under natural conditions. The O_2_ dynamics of the intact rhizosphere is investigated as a function of ambient O_2_ levels, irradiance and plant development for emerged and submerged plants. The presented data are used to provide an improved conceptual and quantitative understanding of spatio-temporal O_2_ dynamics in the rhizosphere of young rice plants.

## Materials and methods

### Plant, soil and rhizotrons

**Fig. 1:**
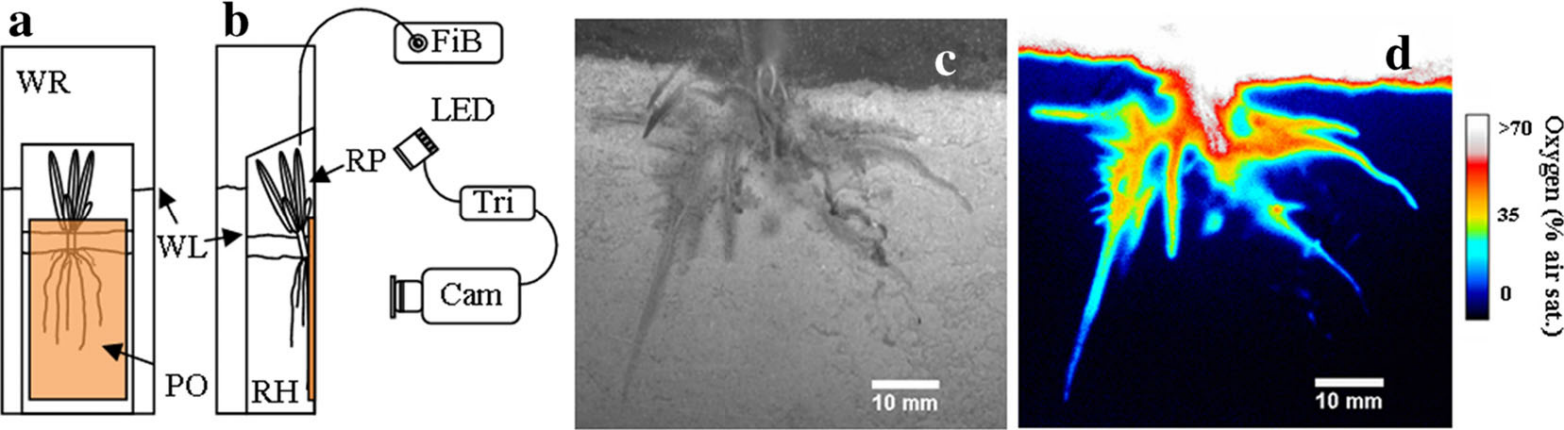
**a** Front and **b** side view of experimental arrangement. Cam; PCO SensiCam camera equipped with longpass emission filter (590 nm), with output to PC, LED; Excitation unit with high power LED and excitation filter, RH; Rhizotron box with plant and soil, WR; water reservoir, PO; O_2_ sensitive planar optode foil, RP; rice plant, Tri; trigger/timing unit with trigger input to PC and LED, WL; water level, FiB; fiber optic O_2_ meter for measurements in air/water above the plant. During optode imaging the water reservoir and the rhizotron was kept at an inclination of 45°. The water level in the rhizotron shown is for an emerged plant. Schematic not to scale. **c** Black and white image of a typical rice root as seen through the transparent rhizotron window, membrane and planar optode. **d** Planar optode O_2_ image of the rhizosphere of rice (*Oryza sativa* L.) from the plant show in panel **b**. The image is scaled to only show O_2_ levels from 0 to 70 % air sat. for better visualization of the rhizosphere associated O_2_ dynamics

Acrylic rhizotrons (H × W × D: 40 × 10 × 1.5 cm) (Fig. 1a, b) were packed with soil sieved to < 2 mm. The soil was loamy sand (sand; 510 g kg^−1^ sand, 420 g kg^−1^ silt, 70 g kg^−1^ clay; pH_(CaCl2)_ 5.4; 23 g kg^−1^ organic matter). The soil was added in layers and was carefully compacted to ensure an overall homogeneous soil structure. The porosity of the soil was calculated based on the mass of soil filled in the rhizotrons, the filled rhizotron volume and estimated soil particle density of 2.65 g cm^−1^. The depth of the soil was ~25 cm. A detachable front window of the rhizotron was opened and the soil surface was covered by a sheet of transparent Nuclepore membrane (Whatman.com, 0.2 μm pore size), ensuring that the soil and roots remained undisturbed during removal of the front window. The membrane did not have any influence on the planar optode imaging - this was confirmed by comparing calibration curves for the planar optode with and without the membrane (data not shown). Subsequently, the rhizotrons were gently filled with tap water and kept anaerobic for two weeks prior to transplanting the rice seedlings.

Rice seeds, *Oryza sativa* (*Indica* cv. RIL 46) were germinated for ten days between moist tissue paper before transplanting the seedlings into the anaerobic soil. At the time of transplantation the roots and shoots were ~10 and 8 mm long, respectively. Seedlings were planted close to the front window of the rhizotrons (Fig. 1b), which were kept at an inclination of 45° to ensure that roots developed along the front window of the rhizotron.

Plants were grown at 22 ± 2°C and an irradiance of ~150–200 μmol photons m^−2^ s^−1^ with a 14:10 h light:dark cycle for three weeks prior to the three weeks long experimental period. The soil was kept in fully water saturated and anaerobic conditions. However, during the experimental period, the water height inside the rhizotron was varied to simulate both emerged and submerged conditions for the rice plants. The water level was maintained by replacing the evaporated water. No nutrients were added to the system.

In the following sections we use the term “emerged” for plants growing with leaves exposed to the air, but with a water height of 8–10 cm inside the rhizotron, covering the majority of the stem (Fig. 1 a, b). For “submerged” plants the entire plants were covered by water. During plant growth and the experimental period, the lower part of the rhizotron was kept in darkness to avoid exposing the roots and soil to light, which could have affected the root development and induce the growth of microphytes at the front plate.

Before planar optode imaging, the front window of the rhizotron was removed and the rhizotron was transferred into a larger water reservoir, with the root and soil surface facing the front of the reservoir (Fig. 1b). To ensure that the soil experienced minimal O_2_ exposure as the front window was removed, the procedure was carried out in a deoxygenated water basin. During the above procedure the rhizotron was kept at an inclination below 45°, to ensure minimal displacement of the soil and root. The rhizotron was fastened against the wall of the reservoir chamber by a number of braces. The transparent front window of the reservoir chamber had previously been equipped with an O_2_ sensitive planar optode foil (see later). The replacement of the front window did not lead to any disturbance or oxygenation of the soil as evaluated from the planar O_2_ sensor. Furthermore, the soil and plants were left to stabilize for a minimum of 24 h to ensure that all O_2_ gradients had reestablished before the experimental work was initiated.

The water level in the reservoir was maintained below the top of the rhizotron (Fig. 1a). The water reservoir with rhizotron was then placed in front of the camera and LED setup. During O_2_ imaging, the water reservoir including the rhizotron was kept at a 45° inclination with the O_2_ imaging camera positioned perpendicularly to the optode. The long term stability of planar optodes sensors, similar to the ones used in this study, has been reported to be in the order of months (Precht et al. 2004) and we likewise observed no drift in the signal. No toxic effects of planar optodes have been reported.

### Optode sensor fabrication

The O_2_ quenchable luminophore PtTFPP (Platinum(II)-5,10,15,20-tetrakis-(2,3,4,5,6-pentafluorphenyl)-porphyrin (frontiersci.com), was chosen as O_2_ indicator due to its excellent photostability and relatively long luminescent lifetime (Borisov and Klimant 2007). Coumarin C545 (SigmaAldrich.com) was used as antenna dye in order to increase the brightness of the indicator (Mayr et al. 2009; Larsen et al. 2011). PtTFPP and Coumarin C545 were mixed in a 1/2 % (wt/wt) ratio, respectively, and dissolved in 4 % (wt/wt) polystyrene to form a sensor cocktail, using chloroform as solvent. The sensor cocktail was coated onto a 125 μm thick transparent polyester foil (Goodfellow.com) using a homemade knife-coating device. The final thickness of the highly transparent O_2_ sensing layer was ~5 μm. The size of the optode was 8 × 15 cm and covered the entire rhizosphere of the rice plant (Fig. 1a).

### Rhizosphere imaging of O_2_ and plaque

The planar optode system used in this study is similar to what has been presented previously (Holst et al. 1998; Frederiksen and Glud 2006) and will only be described briefly. The luminescent lifetime of the O_2_ quenchable luminophore was recorded with a fast gateable 12-bit Charged Coupled Device camera (SensiCam, PCO.de) through a 590 nm longpass filter (UQGoptics.com). Excitation light was delivered from four blue high power LEDs (LXHL-LR3C, Luxeon.com) equipped with a 470 nm short pass filter (UQGoptics.com). The operation of camera and LED’s were synchronized via a custom made PC-controlled triggerbox by the software Look@Molli (Holst and Grunwald 2001). The luminescent lifetime was inferred from two well defined time frames configured by the software. Recorded images were calibrated using the luminescent lifetime for two known O_2_ levels (saturation in the overlying water and 0 % air sat. in anoxic soil), using a modified Stern-Volmer equation (Eq. 1) (Klimant et al. 1995; Glud et al. 1996)

1 $$ \frac{\tau }{\tau_0}=\left[\alpha +\left(1-\alpha \right)\left({\scriptscriptstyle \frac{1}{1+{K}_{SV}\cdot C}}\right)\right] $$

Where τ_0_ is the luminescent lifetime in the absence of O_2_ and τ is the lifetime in the presence of O_2_ at levels (C), K_SV_ the Stern-Volmer quenching constant and α is the non-quenchable fraction of the luminescent signal, the latter experientially determined to 0.12. All images were recorded in darkness, i.e., for “light-images” ambient light was briefly turned off, to avoid interference from ambient light. For each O_2_ image a corresponding black and white (BW) image was recorded. The maximum theoretical spatial resolution that could be achieved with the given optical configuration was ~120 × 120 μm, however, we estimate that the effective spatial resolution is two-three times lower that the maximum theoretical resolution due to light guidance in sensor foil and acrylic walls (Fischer and Wenzhofer 2010). This, however, will not affect any of the qualitative or quantitative conclusions made in this study.

The diffusive O_2_ uptake (DOU) just below the SWI was calculated from extracted vertical O_2_ profiles, using Fick’s first law of diffusion (Jørgensen and Revsbech 1985); DOU = ϕ × Ds × ∂C/∂z, where ϕ represent the soil porosity, D_s_ the molecular diffusion coefficient of O_2_ in the soil at the given temperature and ∂C/∂z represent the O_2_ gradient. The average volume-specific O_2_ consumption was calculated as DOU/O_2_ penetration depth (Glud 2008). In the following sections we define the rhizosphere oxic area as areas with O_2_ levels above the detection limit of the optode sensor ≥ 1 % air sat. (2.3 μmol L^−1^). The detection limit is quantified as three times the standard deviation of the measured concentration at anoxia for an area of 5 × 5 cm. We predominantly use the “oxic area” as a parameter for describing the rhizosphere oxygenation as it allow for a direct comparison between plants and treatments without converting the values to a more complex geometry. However, the oxic area is directly related to the ROL. As only one planar optode imaging system was available, not all plants were imaged continuously during the 3 week experimental period; hence, the longest time series recorded is 6 days. The two-dimensional distribution of plaque formation in the rhizosphere was imaged in a parallel, experiment. Due to overlapping absorption spectra of blue light by the plaque and planar optode, it was not technically possibly to perform simultaneously imaging and hence plaque imaging was performed on a separate and older root system. The imaging principle is based on blue light absorption of Fe- and Mn-oxides (Sherman and Waite 1985; Estapa et al. 2012), the dominating minerals of root plaque in aquatic environments (Christensen and Sand-Jensen 1998). The absorption was imaged using a modified Canon D1000 camera (Larsen et al. 2011) and a blue LED light source, as previously described for the planar optode imaging.

### Experimental procedure

The O_2_ distribution in the rhizosphere was investigated as function of irradiance and O_2_ levels in the overlying water and air, respectively, for both submerged and emerged plants. The O_2_ saturation was regulated by mixing N_2_ and atmospheric air by means of two 5850S mass flow controllers (BrooksInstruments.com) controlled by a type 0154 digital control/readout unit (BrooksInstrument.com). The O_2_ saturation in the rhizotron above the soil level was monitored in parallel by a Microx TX3 micro-optode O_2_ meter (PreSens.de). Levels of irradiance were manipulated by varying the height of the growth lamp (400 W HQI) relative to the plant, the absolute irradiance was measured with a Li-Cor 250 light meter equipped with a planar LI-190 Quantum Sensor (Licor.com). In total three plants were investigated.

## Results

### Soil and root O_2_ dynamics

The soil in the rhizotron had a porosity of 0.58. The O_2_ penetration depth at the soil water interface (SWI) was 2.7 mm (SD ± 0.2, *n* = 6) at 100 % air sat. (273 μmol L^−1^), as derived from the planar optode images. The diffusive O_2_ uptake (DOU) just below the SWI was 17.5 mmol m^−2^ d^−1^ (SD ± 2.9, *n* = 6). The DOU represents the soil O_2_ uptake meditated by diffusion (Glud 2008). The average volume-specific O_2_ consumption amounted to 0.07 nmol cm^−3^ s^−1^ (SD ± 0.015, *n* = 6). This value was used to estimate the integrated rhizosphere related O_2_ consumption rates (see later). The O_2_ penetration depth and volume-specific O_2_ consumption rates are in agreement with values measured in natural rice paddy soil, ranging between 0.8 to 2.0 mm and ~0.02–0.14 nmol cm^−3^ s^−1^, respectively (Damgaard et al. 1998; Reim et al. 2012).

Root system architecture and the corresponding O_2_ images were investigated for three rhizotrons each containing one 3–6 week old plant; a typical example is depicted in Fig. 1c, d. Typically, a zone of high O_2_ levels was observed at the base of the plant (later referred to as ‘basal root zone’) that was usually connected to the O_2_ rich overlying water. This basal root zone was observed to have a high density of small young roots. From the central oxygenated basal root zone, 4–7 roots surrounded by a millimeter-thin oxic zone could be observed, extending into the otherwise anoxic soil. The oxic zone surrounding these roots generally embedded the entire length of the root axis. The deepest roots and associated oxygenated zone were observed 81 mm below the SWI. From the BW images obtained in parallel to the O_2_ images, an average root diameter of ~0.32 mm was estimated for root section behind the conically-shaped root tip; this value corresponds well with other estimates from the literature of 0.35 to 0.5 mm for rice plants of similar age (Revsbech et al. 1999; Deng et al. 2010).

All investigated plants kept large parts of the soil oxic throughout the three-week experimental period. For the three plants the oxic area ranged from 631 to 1080 mm^2^ at ambient O_2_ levels of 100 % air sat. and irradiance of ~200 umol photons m^−2^ s^−1^ throughout the experiential period.

Most roots were leaking O_2_ however 5–10 % of the actively growing did not show any O_2_ leakage. There was no apparent correlation between depth in the soil and the intensity of O_2_ leakage.

### Irradiance induced O_2_ dynamics in the rhizosphere

**Fig. 2:**
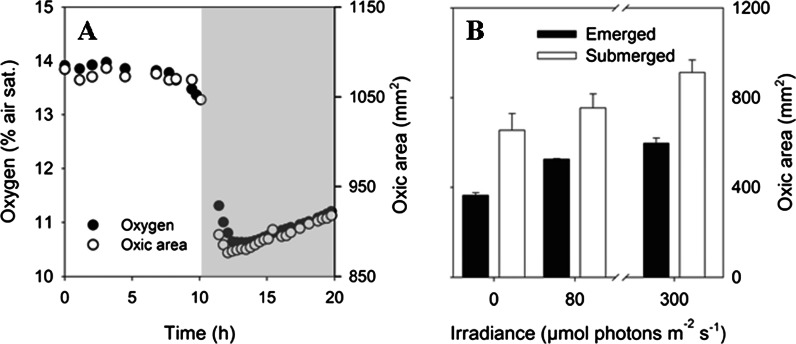
**a** Temporal development of the mean O_2_ level (*black circle*) and the oxic area (*white circle*) in the rhizosphere of a rice plant during a light/dark transition for an emerged plant (see text for explanation). The oxic area was defined as the area with O_2_ levels >1 % air sat. Light grey color represent the dark period. The O_2_ levels represent the mean value within the corresponding oxic area and are based on measurements of 60,763 to 76,388 pixels. **b** Development of the oxic area in the rhizosphere as function of irradiance for an emerged (*black bars*) and submerged plant (*white bars*). The difference in oxic area for the two plants is due to variation in plant size. *Error bars* represent SD, calculated form three individual images at each irradiance level. Each image is based on measurements of 25,294 to 41,502 pixels

Light availability affected the O_2_ level in the rhizosphere for both emerged and submerged plants. For an emerged plant the onset of darkness lead to a rapid decrease in the oxic rhizosphere area of 19.2 % (Fig. 2a) from a stable value of 1080 mm^2^ in light, to a minimum of 876 mm^2^ ~ 2 h after the onset of darkness. The decrease in O_2_ was closely matched by a simultaneous decrease of 24.3 % in the mean O_2_ level within the oxic area. After the initial decrease the O_2_ availability increased during the prolonged exposures to darkness (Fig. 2a). During a period of ~8 h the absolute increase in total rhizosphere oxic area and mean O_2_ level, was found to be 41.8 mm^2^ and 0.51 % air sat., corresponding to an increase of 4.77 and 4.80 %, respectively. The trend of increasing O_2_ levels during darkness was observed for several day-night transitions and all plants, but the changes differed for different sections of the rhizospheres. The mean O_2_ level at the basal root zone and at root tips was observed to increase by 0.17–0.19 % air sat. h^−1^. In contrast single roots showed only an increase of ~0.04 % air sat. h^−1^.

For an emerged plant a step-wise increase in irradiance from 0 to ~300 μmol photons m^−2^ s^−1^ caused a gradual increase in rhizosphere oxic area of 64.0 % (from 364 to 597 mm^2^), the corresponding increase in the mean O_2_ level within the oxic area was 57.7 % (Fig. 2b). As for the day-night transition roots and root tips showed smaller change in O_2_ level, relative to the basal root zone when levels of irradiance was changed. During these experiments the O_2_ level in the air above the emerged plant was maintained at 100 % air sat. Changing the irradiance level for a submerged plant gave similar results to those of emerged plants (Fig. 2b). It should be noted that the oxic area of the submerged plant was larger compared to the emerged plant due to difference in plant size, the trend and magnitude of the increase in oxic area is however comparable between the plants. The increase in oxic area for the submerged plant was 39.2 % when changing the irradiance from 0 to 300 μmol photons m^−2^ s^−1^.

### O_2_ induced dynamics in the rhizosphere

**Fig. 3:**
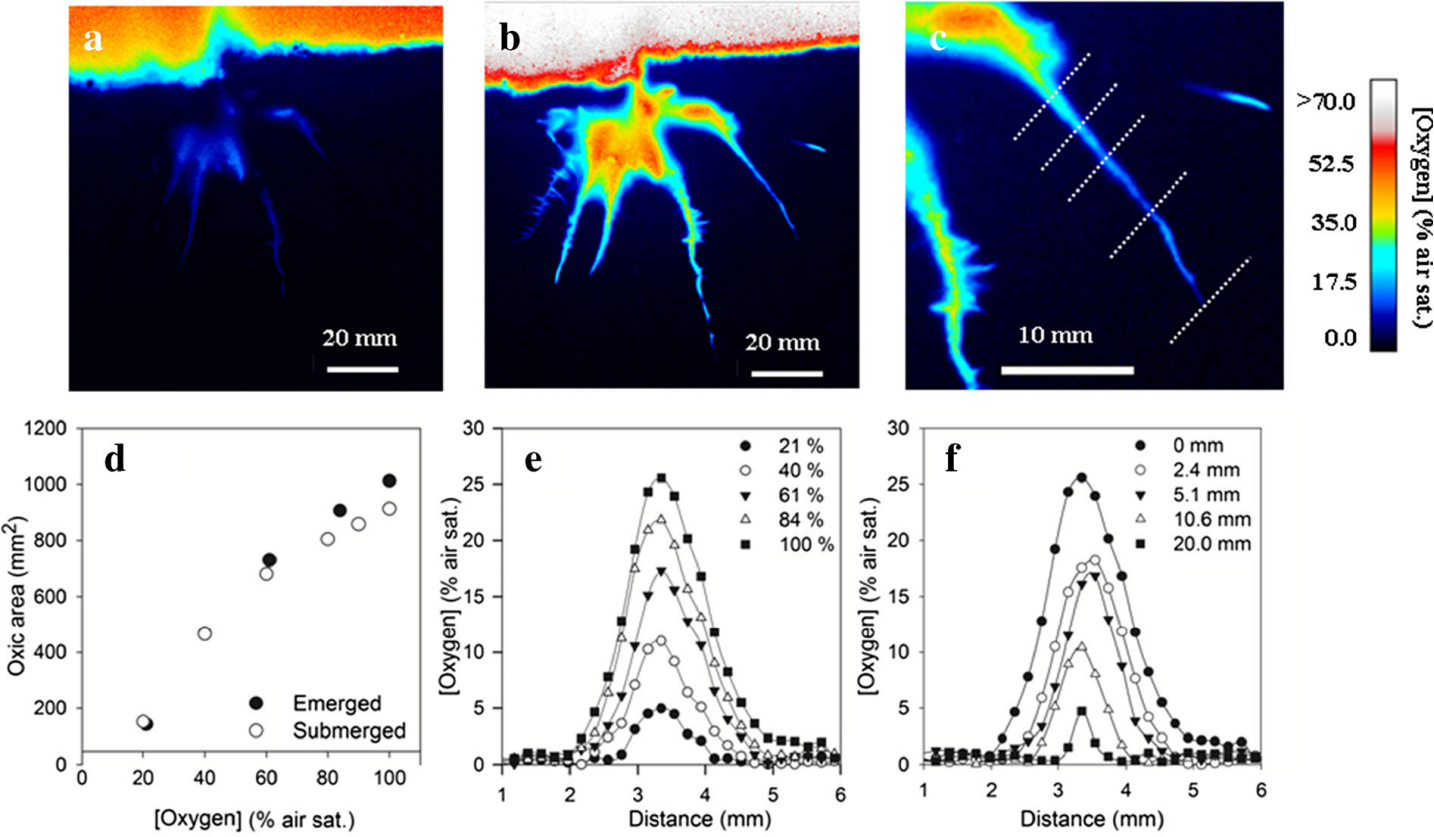
**a** Planar optode O_2_ images of the rhizosphere of emerged rice (*Oryza sativa* L.) as function of O_2_ level in the air above the plant. The O_2_ level in the air is 21 and 100 % air sat., for (**a**) and (**b**) respectively. **c** Close-up of a single root at 100 % air sat. in the air above the plant. The *dashed lines* represents the position of extracted profiles, presented in (**e**) and (**f**). Images are scaled only to show O_2_ levels from 0 to 70 % air sat., for better visualization of rhizosphere O_2_ dynamics. **d** Oxic area of the rhizosphere as function of O_2_ level in the air and water above the plant for a submerged and emerged plant. **e** Variability of O_2_ distribution across a single root from one profile, as function of O_2_ level in the air above the plant. The position of the extracted profiles is equivalent to the “0 mm” profile (furthest away from the tip on panel **c**). **f** Spatial variability in root O_2_ distribution as function of distance from base of the root towards the tip, the position of the profiles are indicated on (**c**), with profile “20 mm” closest to the root tip. The points in panel **e** and **f** represent individual pixels, with a spatial resolution of 120 × 120 μm

Even though irradiance had considerable effect on the O_2_ levels and dynamics in the rhizosphere, the most dramatic changes were induced by regulating the O_2_ level in the air or water surrounding the aboveground plant (Fig. 3a, b and d). For an emerged plant exposed to 100 % air sat., the root oxic area was 1012 mm^2^. However as the O_2_ level was stepwise lowered, the anoxic area gradually increased and the oxic area only accounted for 142 mm^2^ at ambient O_2_ levels of 21 % air sat. (Fig 3d), corresponding to a decrease in the oxic area of 870 mm^2^. The maximum O_2_ level in the oxic area decreased from 88.2 to 18.9 % air sat., respectively, whereas the mean O_2_ level in the oxic area decreased from 34.8 to 12.7 % air sat. Extrapolation of the observed trends in oxic area and average O_2_ levels indicated that the rhizosphere would become completely anoxic at ambient O_2_ levels of ~13 % air sat. The root tips appeared to be most sensitive to declining ambient O_2_ levels; here anoxia was reached at ambient levels of 39.4 % air sat. (±SD = 10.7, *n* = 5). In comparison anoxia was reached at ambient O_2_ levels of 19.7 % and 13.7 % (±SD = 10.6, *n* = 4), for the basal root zone and the middle section of long roots, respectively.

Extracted O_2_ profiles from a typical root (Fig 3e), demonstrated that the O_2_ level at the root surface decreased from 26.2 to 5.14 % air sat., when the O_2_ level in the ambient air was reduced from 100 to 21 % air sat. Consequently, the width of the oxic zone decreased from 1.3 to 0.6 mm. The extent of the oxygenated area varied between neighbouring roots and along individual roots. A transect of O_2_ profiles perpendicular to a single root is presented in Fig. 3f, showing that the width of the oxic zone decreased from 2.6 to 0.3 mm when approaching the root tip at 100 % air sat., and irradiance of ~200 μmol photons m^−2^ s^−1^. The general pattern as shown in Fig. 3e and f were observed for all roots on the plant and for all plants investigated.

Changing the O_2_ level in the ambient water for submerged plants gave very similar results as for emerge plants. In fact we found that the root oxic area disappears when an O_2_ level in the water was reduced to ~13 % air sat., this was the same threshold as estimated for the emerged plant (Fig. 3d).

### Temporal O_2_ dynamics

**Fig. 4:**
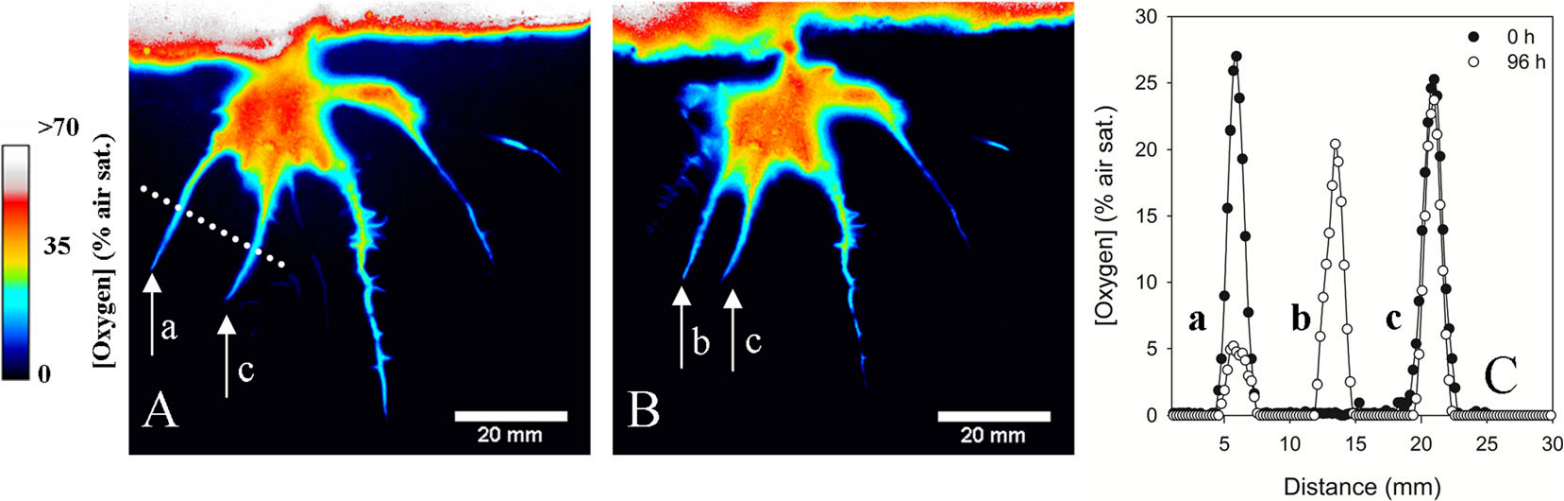
Temporal development of O_2_ in the rhizosphere of a rice plant. **a**, **b** O_2_ images recorded with 92 h interval from a 180 h long time series. It can be observed that as one root (arrow ‘a’) experience a dramatically reduction in the oxic area during the 92 h period, a new oxic zone develops around a fast growing neighbouring root (arrow ‘b’, growth rate 2.6 mm d^−1^). The oxic area around the roots to the right “c” remains unchanged. Extracted O_2_ profiles from image (**a**) and (**b**) are show in panel (**c**), the position of the profile is indicate on the images as a dotted line. A close-up of root “a” with visible leakage from lateral roots is shown in Fig. 5. The points in panel **e** and **f** represent individual pixels, with a spatial resolution of 120 × 120 μm

**Fig. 5:**
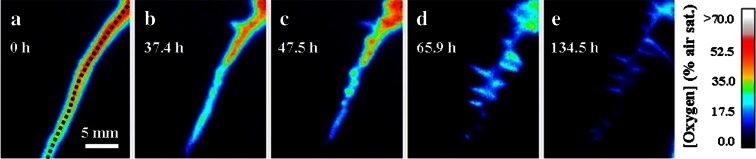
Temporal development of ROL in the rhizosphere of a single rice root during a ~5.5 d period. **a**, **b**, **c**, **d** and **e** represent a close up of the root “a” in Fig. 4. The position of the root is indicated with the dashed line in panel **a**. From the time series it can be observed how ROL from the main root gradually disappears, presumably due to a formation of a barrier to ROL. Simultaneously it can be observed how multiple fine lateral roots are starting to leak O_2_

The spatial extent of the oxygenated rhizosphere zone was dynamic, as the rhizosphere evolved; older roots ceased to leak O_2_, while young, newly grown roots exhibited high O_2_ release. We observed root growth rates of up to 2.6 mm d^−1^. One example of the temporal development is depicted in Fig. 4, where an emerging root begins leaking O_2_ into previously anoxic soil (root ‘a’ in Fig. 4), while concurrently the O_2_ levels around a neighbouring root drastically diminish (root ‘b’). Interestingly it could be observed that as O_2_ leakage from the main part of root ‘a’ diminishes with time, leakage from its laterals roots could be observed (Fig. 5). In contrast, O_2_ release from the neighbouring roots remained virtually unchanged during this ~5.5 day period.

### Plaque distribution

**Fig. 6:**
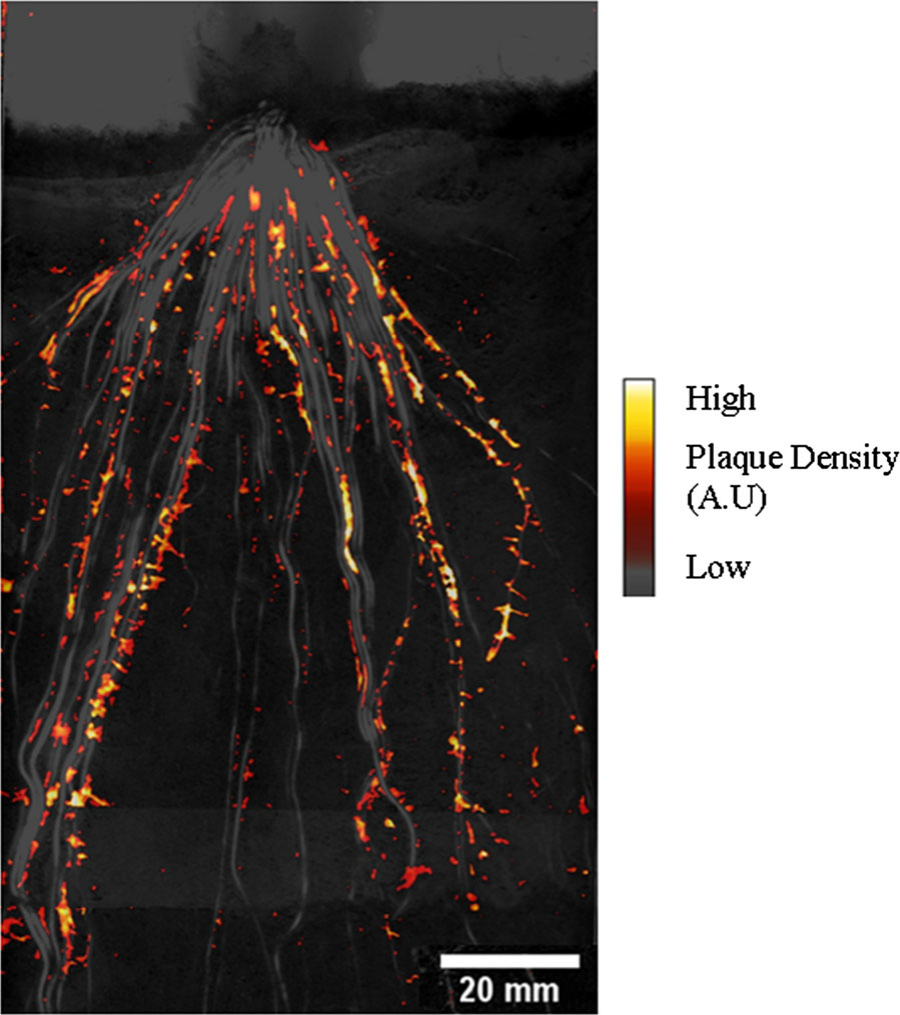
High resolution two-dimensional visualization of metal plaque on rice roots growing in submerged soil – the metal plaque image is projected onto a black and white image of the root position. A high spatial variability of the plaque among the individual roots can be observed. Imaging can be performed continuously in real time and allows for studies of spatial and temporal developments of plaque. Areas with high densities of plaque are shown with bright colors A.U. (Arbitrary units)

The distribution of metal plaques was highly patchy and variable between different roots – no roots were observed to have plaque formation along the entire root (Fig. 6). The total visual metal plaque-containing area in the rhizosphere was found to be 739 mm^2^, 5.9 % of the visible area. Plaque density was highest in a zone 41.1–66.1 mm below the soil surface. Below this zone, plaque density decreased gradually. The soil area covered by plaque did on average corresponded to ~30.4 % of the visible root surface area.

## Discussion

### O_2_ dynamics in the rhizosphere studied by planar optodes

We present novel high resolution two-dimensional images of the O_2_ dynamics of the rhizosphere of lowland rice. We demonstrate that the spatio-temporal O_2_ distribution in the rhizosphere is highly dynamic and affected by abiotic factors (light and ambient O_2_ level) and different responses are observed for different sections of the rhizosphere. The use of high resolution planar optodes greatly improves the possibility of understanding and mapping these dynamics and furthermore underpin that single point or single profile measurements, cannot be extrapolated to the entire rhizosphere.

However, all planar optode imaging is performed along a wall, this will have implications for the measured concentration and distribution of O_2_ (Wenzhofer and Glud 2004; Glud 2008; Meysman et al. 2010). As the wall does not consume O_2_, the oxic zone surrounding the roots is wider than for roots only surrounded by soil. However, assuming that the oxic zone around roots along the wall can be approximated as half-spherical or half-cylinders and that the total oxic volume around roots are independent of the wall, the radial O_2_ distribution for” undisturbed” roots can be calculated (Frederiksen and Glud 2006; Polerecky et al. 2006). The corrected width of the oxic zone is 29 to 41 % less (depending on the width of the oxic zone) than the width observed at the planar optodes. This correction is used in all the following calculations.

### Influence of irradiance and ambient O_2_ levels on rhizosphere O_2_ dynamics

The O_2_ release from the roots was clearly affected by changes in irradiance levels – suggesting that photosynthetic O_2_ production is important for regulating radial O_2_ loss (ROL). The observed mean increase of 57.7 % air sat. in the rhizosphere O_2_ level during an increase in irradiance from 0 to 300 μmol photons m^−2^ s^−1^, is however lower than reported values from microsensors studies for submerged rice grown in artificial substrates. One study found an O_2_ increase in rice root tissue 10 mm behind the root tip of ~ 500 % during a dark–light cycle with irradiance: 350–400 μmol photons m^−2^ s^−1^ (Colmer and Pedersen 2008). These values however represent measurements inside the tissue of plants grown in non-reducing agar medium. Likewise it have been reported that a dark–light transition (irradiance: ~900 μmol photons m^−2^ s^−1^), increased the O_2_ in the root tissue by >300 %, for rice plants grown in nutrient solution (Waters et al. 1989). Only a few studies have, as the present one, investigated rhizosphere O_2_ dynamics of plants growing in natural soil. One microsensor study, found no O_2_ changes inside root tissues during a dark–light cycle (irradiance: 400–500 μmol photons m^−2^ s^−1^) for rice plants of similar age compared to the plants in our study (Revsbech et al. 1999). The explanation for this large discrepancy among studies is not clear, but it is apparent that the highest responses to light–dark shifts apparently are found for plants grown in artificial, non-reducing media and are largely based on single point measurements with O_2_ electrodes (Waters et al. 1989; Colmer and Pedersen 2008). One explanation is that comparison between studies is confounded by differences in plant morphology e.g., ROL barrier and experimental conditions of the respective studies. However, the variability of ROL observed in this study show that single-point measurements only give a limited insight in the true spatial heterogeneity, as large differences in the extent of rhizosphere oxygenation may exist within a single root system. In fact, analyzing the dark–light response (irradiance: 300 μmol photons m^−2^ s^−1^) of individual pixels from a single root, we found an relative increase up to 195 %, while the average value across the oxygenated area of this individual root was only 31.2 %.

After the initial sharp drop in rhizosphere O_2_ at the onset of darkness, the subsequent steady increase in rhizosphere O_2_ levels and expanding oxic area (Fig. 2a) must be associated to I) concurrent reduction in root (and shoot) respiration and/or II) an alternative reduction in O_2_ respiration of microorganisms in the surrounding soil. The first point is supported by the evidence of reduced dark respiration in roots following substrate depletion in plant tissue after prolonged exposure to darkness (Waters et al. 1989; Colmer and Pedersen 2008). As the metabolic activity is highest in root tips, it is to be expected that shortage of substrates would occur there first. This is consistent with our finding that root tips and the basal root zone, hosting relatively large amounts of young metabolically active tissue, exhibit the fastest increase in O_2_ levels after the initial decline. Therefore, we suggest that reduced respiration in the root tissues, at least partly, could explain the observed increase of ROL during darkness. Likewise, reduced leakage of organic substrates from the root tip area would reduce soil metabolism and increase the ambient O_2_ availability during nighttime.

**Fig. 7:**
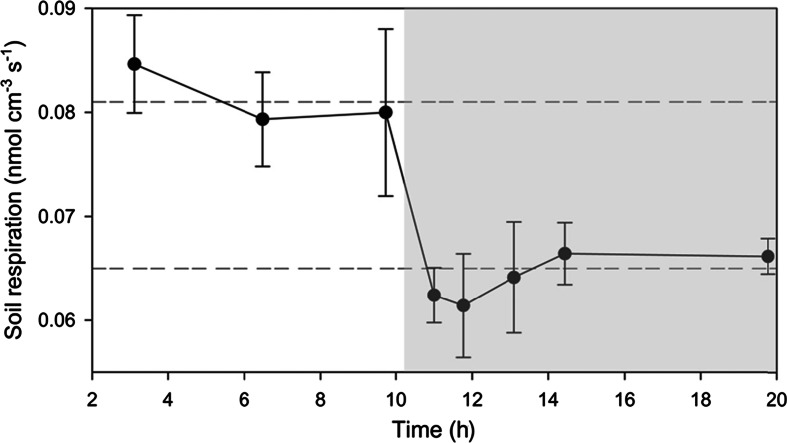
Volume specific O_2_ respiration at a typical root tip. Rates are estimated based on extracted concentration profiles across the tip. The rates are calculated based on simple planar diffusion geometry. Values are mean for both “sides” of the root tip, with error bars representing ± SD, *n* = 2. The grey shaded area represents the dark period. The upper and lowers horizontal dashed lines represent the mean O_2_ respiration for the light and dark period, respectively (Light: 0.081 nmol cm^−3^ s^−1^, ±SD = 0.5 × 10^−2^, *n* = 6; Dark: 0.064 nmol cm^−3^ s^−1^ ± SD = 0.96 × 10^−2^, *n* = 10). In comparison, the soil volume specific respiration as calculated for the primary soil-water interface was found to amount to 0.07 nmol cm^−3^ s^−1^ ± SD = 0.015, *n* = 6

We cannot quantitatively discriminate between the importance of the two processes, but we suggest that the resolved dynamic is related to reduced O_2_ consumption in both the root and the surrounding soil. Both factors are presumably being regulated by the availability of plant derived exudates that in some cases have been demonstrated to decrease during dark periods (Watt and Evans 1999; Badri et al. 2010). The reduction in soil/root respiration, estimated from extracted O_2_ profiles, during a day-night transition amounted to 32 %, from 0.081 nmol cm^−3^ s^−1^ (±SD = 0.5 × 10^−2^, *n* = 6) to 0.064 nmol cm^−3^ s^−1^ (±SD = 0.96 × 10^−2^, *n* = 12) (Fig. 7).

### Regulation of rhizosphere O_2_ dynamics

Variation in the O_2_ levels in the ambient air/water surrounding the plant imposed the largest O_2_ dynamics in the rhizosphere. For the plants investigated it apparently made little difference in rhizosphere O_2_ levels if plants were submerged or emerged. We do not know if the rice plants in our study had leaf gas film, previous studies have shown that this is genotypic dependent. However, the existence of such gas film could explain the apparent tolerance to submergence, as it would facilitate an efficient gas exchange between plant and water (Pedersen et al. 2009).

The net downward O_2_ transport is passive, and the degree of ROL is mainly controlled by the plants ability to adapt root morphology e.g., by elevated levels of suberin in the exodermis, lignin in the sclerenchyma and increased aerenchyma volume (Kotula et al. 2009). The rapid decrease in root O_2_ leakage from the main root axis and subsequently O_2_ leakage from the fine lateral roots, under otherwise constant environmental conditions (Figs. 4 and 5), most likely reflect a shift in root morphology and formation of a barrier to ROL. Such rapid development of a barrier to ROL have previously only been observed for roots transferred from oxic to anoxic conditions (Insalud et al. 2006). To our knowledge, the transition in O_2_ leakage from root axis to fine lateral roots has not previously been observed. However, capturing such transition is only achievable applying high resolution imaging approaches. From the BW images it was not possible to conclude if laterals were present already at Fig. 5a “0 h” or if laterals were formed during the image sequence. It have been proposed that fine lateral roots are the dominant surface for nutrient uptake (and remain permeable to O_2_) (Kirk 2003), thus it is plausible that the observed transition also reflects a change in root functioning.

The present study shows that ROL of root tips was particular sensitive to the ambient O_2_ level experienced by the aboveground plant, presumably as a result of intensified metabolism, longer transport distance from the soil surface and a large surface-to-volume ratio.

### Rhizosphere O_2_ budget

Quantitative ROL rates can be calculated as : *J* = *ϕ* × *R*_*S*_ × *L* × (*L*/2 × *A* + 1), where *ϕ* is the soil porosity, R_s_ the soil volume specific O_2_ uptake, L equals half the width of the oxic zone (corrected for radial geometry, see above) and A is the root diameter (Fenchel 1996). We used an average root diameter of ~0.32 mm as assessed from 6 images and 4 different roots and the value for R_s_ calculated from the soil-water interface (see above). The calculated ROL exhibited considerable spatial variability. For instance did the ROL, as calculated from the profiles in Fig. 3c, f, range spatially from 10.6 to 0.46 mmol m^−2^ d^−1^, with an average of 5.22 mmol m^−2^ d^−1^. Extrapolating this procedure to all observed single roots of the image in Fig. 3, (at ~200 μmol photons m^−2^ s^−1^ and 100 % air sat.), provided an mean ROL of 7.7 mmol m^−2^ d^−1^ (*n* = 259 profiles) for all visible roots in Fig. 3b. Although not all roots are accessible for analysis (as some roots must grow in the interior of the rhizotron) a plant specific ROL of 0.27 mmol d^−1^ can be calculated using previously estimated plant specific root surface area of 3.5 × 10^−2^ m^−2^ plant^−1^ (Miyamoto et al. 2001). These findings are comparable to previously reported ROL values of 0.24 to 1.11 mmol d^−1^ for 3 to 6 weeks old rice plants grown in soil (Revsbech et al. 1999; Cheng et al. 2014), but are substantially higher than rates derived from rice plants grown in stagnant hydroponics (Deng et al. 2010; Wu et al. 2012) with ROL rates of 1.3 × 10^−2^ and 2.3 × 10^−2^ mmol d^−1^, respectively. It should be noted that calculations by other authors (Revsbech et al. 1999) and our present study, assume that oxic zones of individual roots do not merge. As can be observed on Figs. 1 and 3, that is not the case at the central root base and the assessments should thus be regarded as a maximum value.

Assuming densities of 40 to 100 plants m^−2^ (as found in typical paddy rice fields (Xu et al. 2006)), root induced O_2_ leakage accounts for 10.8 to 27.0 mmol m^−2^ d^−1^, at 100 % air saturation in the ambient air/water and at an irradiance of ~200 μmol photons m^−2^ s^−1^. Corresponding night time values would be 8.7 to 21.8 mmol m^−2^ d^−1^. Calculated on a 24 h time scale (14:10 h, light–dark cycle) total soil O_2_ uptake in the rhizosphere amount to 144 % of the diffusive O_2_ uptake at the soil-water interface, indicating a considerable rhizosphere-mediated gas exchange in rice paddies.

Altogether, our rates compare well to previously reported values with daytime O_2_ uptake in the order of 30 mmol m^−2^ d^−1^ (125 plants m^−2^) (Revsbech et al. 1999).

The oxic volume amounts to 14.9 to 18.7 cm^3^ per plant, for night and daytime, respectively, which for natural plant densities scales (40 to 100 plants m^−2^) to 748 to 1870 cm^3^ m^−2^ and 604 to 1511 cm^3^ m^−2^, for day- and nighttime, respectively. Rice paddies are usually exposed to higher light and temperature than applied in our study. Hence, the absolute values on ROL and O_2_ consumption are therefore not directly comparable to most in situ conditions. Nevertheless, we argue that the conclusion on extensive temporal and spatial variations in ROL of rice plants still is valid. In fact, natural conditions with ever changing conditions in light and temperature would favor an even higher degree of variability.

Most roots showed ROL, and only 5–10 % of roots showed no traces of O_2_ leakage – even at maximum light levels and 100 % ambient air saturation. The fact that most roots of young rice leaks O_2_ aligns with previous observations (Armstrong 1994; Revsbech et al. 1999; Williams et al. 2014). It could be speculated that for young small plants with short roots, O_2_ transport at the prevailing conditions is sufficient to ensure O_2_ to the root-tips and that no morphological adaptations to restrain ROL is required or that short roots might not yet be able for form a barrier to ROL owing to the development stage for the tissue (Shiono et al. 2011).

### Metal plaque distribution in the rhizosphere

Metal plaque images showed a high degree of spatial variation, most likely reflecting variations in O_2_ availability (Fig. 6). Plaque formations mainly reflects Fe oxidation and have been considered to be predominately abiotically mediated (Mendelssohn et al. 1995), suggesting a direct link between O_2_ leakage and plaque formation (Wu et al. 2012; Cheng et al. 2014). Our investigations showed that metal plaques were predominantly found in a millimeter wide zone around the roots, corresponding to the observed width of the oxic zone, but were heterogeneously distributed along the root in contrast to the observed continuous oxic zone along roots. However, as root metal plaques can persist in anoxic soil for months (Weiss et al. 2005) a direct temporal link between ROL and plaque distribution at any given time cannot be expected.

We did not measure metal plaque distribution and O_2_ dynamics on the same plant, but the potential of combining real time imaging of O_2_ and metal plaque distribution/dynamics could contribute to the understanding the heterogeneity in plaque dynamics and associated trace metal cycling (Deng et al. 2010; Seyfferth et al. 2010; Cheng et al. 2014).

By applying high resolution O_2_ imaging we were able so resolve how irradiance, ambient O_2_ levels and plant development affected the O_2_ dynamics across the rhizosphere. The data demonstrated that the spatio-temporal dynamic in the rhizosphere of rice grown in submerged soil is substantially larger than previously anticipated. This underpins that extrapolations based on single point measurements within the rhizosphere, should be done with caution, as they likely provide an oversimplified view on the rhizosphere processes. This improved understanding of the magnitude and scale of O_2_ dynamics can help to advance the understanding of the biogeochemical cycling the rice rhizosphere.

In the presented study we used a luminescent lifetime planar optode imaging approach but it should be noted that comparable data quality can be achieved using more simple and inexpensive imaging approaches (Zhu et al. 2006; Larsen et al. 2011; Tschiersch et al. 2011). With such systems high resolution measuremnets of O_2_, pH and pCO_2_ can be realized, greatly enhancing the possibilities to study the spatialy complex rhizosphere. Furthermore, planar optode imaging can be combined with DGT measuremnts (Diffusive Gradient in Thin films) for simultaniously measuremnets of O_2_/pH and trace metal fluxes (Stahl et al. 2012; Williams et al. 2014).

## References

[CR1] Armstrong W (1980) Aeration in higher plants. In: Woolhouse HW (ed) Advances in Botanical Research. Academic Press

[CR2] Armstrong W (1994). Polarographic oxygen eletrodes and their use in plant aeration studies. Proc. R. Soc. Edinb. B Biol Sci.

[CR3] Armstrong J, Armstrong W (2005). Rice: Sulfide-induced barriers to root radial oxygen loss, Fe2+ and water uptake, and lateral root emergence. Ann Bot.

[CR4] Badri DV, Loyola-Vargas VM, Broeckling CD, Vivanco JM (2010). Root secretion of phytochemicals in Arabidopsis is predominantly not influenced by diurnal rhythms. Mol Plant.

[CR5] Blossfeld S, Schreiber CM, Liebsch G, Kuhn AJ, Hinsinger P (2013). Quantitative imaging of rhizosphere pH and CO2 dynamics with planar optodes. Ann Bot.

[CR6] Borisov SM, Klimant I (2007). Ultrabright oxygen optodes based on cyclometalated iridium(III) coumarin complexes. Anal Chem.

[CR7] Cheng H, Wang M, Wong M, Ye Z (2014). Does radial oxygen loss and iron plaque formation on roots alter Cd and Pb uptake and distribution in rice plant tissues?. Plant and Soil.

[CR8] Christensen KK, Sand-Jensen K (1998). Precipitated iron and manganese plaques restrict root uptake of phosphorus in Lobelia dortmanna. Can J Bot.

[CR9] Colmer TD (2003). Aerenchyma and an inducible barrier to radial oxygen loss facilitate root aeration in upland, paddy and deep-water rice (Oryza sativa L.). Ann Bot.

[CR10] Colmer TD, Pedersen O (2008). Oxygen dynamics in submerged rice (Oryza sativa). New Phytol.

[CR11] Colmer TD, Gibberd MR, Wiengweera A, Tinh TK (1998). The barrier to radial oxygen loss from roots of rice (Oryza sativa L.) is induced by growth in stagnant solution. J Exp Bot.

[CR12] Damgaard LR, Revsbech NP, Reichardt W (1998). Use of an oxygen-insensitive microscale biosensor for methane to measure methane concentration profiles in a rice paddy. Appl Environ Microbiol.

[CR13] Deng D, Wu S-C, Wu F-Y, Deng H, Wong M-H (2010). Effects of root anatomy and Fe plaque on arsenic uptake by rice seedlings grown in solution culture. Environ Pollut.

[CR14] Estapa ML, Boss E, Mayer LM, Roesler CS (2012). Role of iron and organic carbon in mass-specific light absorption by particulate matter from Louisiana coastal waters. Limnol Oceanogr.

[CR15] Faget M, Blossfeld S, Von Gillhaußen P, Schurr U, Temperton VM (2013) Disentangling who is who during rhizosphere acidification in root interactions: combining fluorescence with optode techniques. Front Plant Sci 4. doi: 10.3389/fpls.2013.00392 10.3389/fpls.2013.00392 PMC3797519 24137168

[CR16] Fenchel T (1996). Worm burrows and oxic microniches in marine sediments. 1. Spatial and temporal scales. Mar Biol.

[CR17] Fischer JP, Wenzhofer F (2010). A novel planar optode setup for concurrent oxygen and light field imaging: Application to a benthic phototrophic community. Limnol. Oceanogr. Methods.

[CR18] Frederiksen MS, Glud RN (2006). Oxygen dynamics in the rhizosphere of Zostera marina: A two-dimensional planar optode study. Limnol Oceanogr.

[CR19] Frenzel P, Rothfuss F, Conrad R (1992). Oxygen profiles and methane turnover in a flooded rice microcosm. BFS.

[CR20] Glud RN (2008). Oxygen dynamics of marine sediments. Mar. Biol. Res.

[CR21] Glud RN, Ramsing NB, Gundersen JK, Klimant I (1996). Planar optrodes: A new tool for fine scale measurements of two- dimensional O2 distribution in benthic communities. Mar Ecol Prog Ser.

[CR22] Greenway H, Armstrong W, Colmer TD (2006). Conditions leading to high CO2 (>5 kPa) in waterlogged-flooded soils and possible effects on root growth and metabolism. Ann Bot.

[CR23] Holst G, Grunwald B (2001). Luminescence lifetime imaging with transparent oxygen optodes. Sensors Actuators B Chem.

[CR24] Holst G, Kohls O, Klimant I, König B, Kühl M, Richter T (1998). A modular luminescence lifetime imaging system for mapping oxygen distribution in biological samples. Sens Actuators B.

[CR25] Insalud N, Bell RW, Colmer TD, Rerkasem B (2006). Morphological and physiological responses of rice (Oryza sativa) to limited phosphorus supply in aerated and stagnant solution culture. Ann Bot.

[CR26] Jensen SI, Kuhl M, Glud RN, Jorgensen LB, Prieme A (2005). Oxic microzones and radial oxygen loss from roots of Zostera marina. Mar Ecol Prog Ser.

[CR27] Jiang F, Chen X, Luo A (2009) “Iron plaque formation on wetland plants and its influence on phosphorus, calcium and metal uptake.” Aquat Ecol 43(4):879–890

[CR28] Jovanovic Z, Pedersen MØ, Larsen M, Kristensen E, Glud RN (2015). Rhizosphere O2 dynamics in young Zostera marina and Ruppia maritime. Mar Ecol Prog Ser.

[CR29] Jørgensen BB, Revsbech NP (1985). Diffusive boundary-layers and the oxygen-uptake of sediments and detritus. Limnol Oceanogr.

[CR30] Kirk GJD, Vries F, Teng P, Metselaar K (1993). Root ventilation, rhizosphere modification, and nutrient uptake by rice. Systems approaches for agricultural development.

[CR31] Kirk GJD (2003). Rice root properties for internal aeration and efficient nutrient acquisition in submerged soil. New Phytol.

[CR32] Kirk GJD, Du LV (1997). Changes in rice root architecture, porosity, and oxygen and proton release under phosphorus deficiency. New Phytol.

[CR33] Klimant I, Meyer V, Kuhl M (1995). Fiber-optic oxygen microsensors, a new tool in aquatic biology. Limnol Oceanogr.

[CR34] Kludze HK, Delaune RD, Patrick WH (1993). Aerenchyma formation and methane and oxygen-exchange in rice. Soil Sci. Soc. Am. J.

[CR35] Kotula L, Ranathunge K, Schreiber L, Steudle E (2009). Functional and chemical comparison of apoplastic barriers to radial oxygen loss in roots of rice (Oryza sativa L.) grown in aerated or deoxygenated solution. J Exp Bot.

[CR36] Larsen M, Borisov SM, Grunwald B, Klimant I, Glud RN (2011). A simple and inexpensive high resolution color ratiometric planar optode imaging approach: application to oxygen and pH sensing. Limnol. Oceanogr. Methods.

[CR37] Mayr T, Borisov SM, Abel T, Enko B, Waich K, Gn M, Klimant I (2009). Light harvesting as a simple and versatile way to enhance brightness of luminescent sensors. Anal Chem.

[CR38] Mendelssohn I, Kleiss B, Wakeley J (1995). Factors controlling the formation of oxidized root channels: a review. Wetlands.

[CR39] Meysman FJR, Galaktionov OS, Glud RN, Middelburg JJ (2010). Oxygen penetration around burrows and roots in aquatic sediments. J Mar Res.

[CR40] Miyamoto N, Steudle E, Hirasawa T, Lafitte R (2001). Hydraulic conductivity of rice roots. J Exp Bot.

[CR41] Mongon J, Konnerup D, Colmer TD, Rerkasem B (2014). Responses of rice to Fe2+ in aerated and stagnant conditions: growth, root porosity and radial oxygen loss barrier. Funct Plant Biol.

[CR42] Pedersen O, Rich SM, Colmer TD (2009). Surviving floods: leaf gas films improve O-2 and CO2 exchange, root aeration, and growth of completely submerged rice. Plant J.

[CR43] Polerecky L, Volkenborn N, Stief P (2006). High temporal resolution oxygen imaging in bioirrigated sediments. Environ Sci Tech.

[CR44] Precht E, Franke U, Polerecky L, Huettel M (2004). Oxygen dynamics in permeable sediments with wave-driven pore water exchange. Limnol Oceanogr.

[CR45] Reim A, Luke C, Krause S, Pratscher J, Frenzel P (2012) One millimetre makes the difference: high-resolution analysis of methane-oxidizing bacteria and their specific activity at the oxic-anoxic interface in a flooded paddy soil. ISME J 6: 2128–2139. doi: http://www.nature.com/ismej/journal/v6/n11/suppinfo/ismej201257s1.html 10.1038/ismej.2012.57 PMC3475382 22695859

[CR46] Revsbech NP, Pedersen O, Reichardt W, Briones A (1999). Microsensor analysis of oxygen and pH in the rice rhizosphere. Biol Fertil Soils.

[CR47] Roger PA, Zimmerman WJ, Lumpkin TA (1993) Microbiological management of wetland rice fields. Marcel Dekker, Inc., 270 Madison Avenue, New York, New York 10016, USA; Marcel Dekker, Inc., Basel, Switzerland

[CR48] Rudolph N, Voss S, Moradi A, Nagl S, Oswald S (2013). Spatio-temporal mapping of local soil pH changes induced by roots of lupin and soft-rush. Plant and Soil.

[CR49] Schreiber CM, Zeng B, Blossfeld S, Rascher U, Kazda M, Schurr U, Hoeltkemeier A, Kuhn AJ (2012). Monitoring rhizospheric pH, oxygen, and organic acid dynamics in two short-time flooded plant species. J. Plant Nutr. Soil Sci.

[CR50] Seyfferth AL, Webb SM, Andrews JC, Fendorf S (2010). Arsenic localization, speciation, and Co-occurrence with iron on rice (Oryza sativa L.) roots having variable Fe coatings. Environ Sci Technol.

[CR51] Sherman DM, Waite TD (1985). Electronic-spectra of Fe-3+ oxides and oxide hydroxides in the near ir to near uv. Am Mineral.

[CR52] Shiono K, Ogawa S, Yamazaki S, Isoda H, Fujimura T, Nakazono M, Colmer TD (2011). Contrasting dynamics of radial O-2-loss barrier induction and aerenchyma formation in rice roots of two lengths. Ann Bot.

[CR53] Stahl H, Warnken KW, Sochaczewski L, Glud RN, Davison W, Zhang H (2012). A combined sensor for simultaneous high resolution 2-D imaging of oxygen and trace metals fluxes. Limnol. Oceanogr. Methods.

[CR54] Tschiersch H, Liebsch G, Stangelmayer A, Borisjuk L, Rolletschek H (2011) Planar Oxygen Sensors for Non Invasive Imaging in Experimental Biology

[CR55] Waters I, Armstrong W, Thompson CJ, Setter TL, Adkins S, Gibbs J, Greenway H (1989). Diurnal changes in radial oxygen loss and ethanol metabolism in roots of submerged and Non-submerged rice seedlings. New Phytol.

[CR56] Watt M, Evans JR (1999). Linking development and determinacy with organic acid efflux from proteoid roots of white lupin grown with low phosphorus and ambient or elevated atmospheric CO2 concentration. Plant Physiol.

[CR57] Weiss JV, Emerson D, Megonigal JP (2005). Rhizosphere iron(III) deposition and reduction in a Juncus effusus L.-dominated wetland. Soil Sci Soc Am J.

[CR58] Wenzhofer F, Glud RN (2004). Small-scale spatial and temporal variability in coastal benthic O-2 dynamics: effects of fauna activity. Limnol Oceanogr.

[CR59] Williams PN, Santner J, Larsen M, Lehto N, Oburger E, Wenzel WW, Glud RN, Davison W, Zhang H (2014). Localised flux-maxima of arsenic, lead and iron around root apices in flooded lowland rice. Environ Sci Technol.

[CR60] Wu C, Ye Z, Li H, Wu S, Deng D, Zhu Y, Wong M (2012). Do radial oxygen loss and external aeration affect iron plaque formation and arsenic accumulation and speciation in rice?. J Exp Bot.

[CR61] Xu Z, Zheng X, Wang Y, Wang Y, Huang Y, Zhu J (2006). Effect of free-air atmospheric CO2 enrichment on dark respiration of rice plants (Oryza sativa L.). Agr Ecosyst Environ.

[CR62] Zhu Q, Aller RC, Fan Y (2006). Two-dimensional pH distributions and dynamics in bioturbated marine sediments. Geochim Cosmochim Acta.

